# 

**Published:** 1877-09

**Authors:** 


### CONTENTS.

### COMMUNICATIONS.

On the Structure and Growth of Dentine—*Chr. Sihler, M. D*............ 361
The Involuntary Action of the Nervous System—*John Caldwell M. D*.. 365
A Remarkable Case—*P. R. Thomas, D. D. S*........ ...................... 374
Mechanical Dentistry at the Meeting of the American Association—*L. P. Haskell*........................................................................ 376
Eruption of Two Inferior Central Incisor Teeth Second Day after Birth, Their Extraction followed by Hemorrhage and Death—*M. Th. David* 378
The Future of Dentistry—*W. H. Morgan, D. D. S*...................... 384
Anæsthetic—*L. G. Noel, D. D. S*........................................ 386
Temporary or Plastic Fillings—*Milton H. Chappell*............... ........ 391

### SELECTIONS.

The Food of the Ancients, and of Modern Infants—*William A. Green, M. D*...... ................................................. 394

### EDITORIAL.

A New Microscope.................... ........................... 400
Attention............................................................401
Removal ............................................................ 402
New Dental Chair......... ........................................ 402
Dental Convention..... ........... ........... .................... 402
American Dental Association............................................ .. 403

